# The impact of COVID‐19 on the mental health of dental students at an Australian school

**DOI:** 10.1111/adj.13029

**Published:** 2024-07-16

**Authors:** S Akhtar, J Tissainayagam, J Lo, A Siddiqi, S Zafar

**Affiliations:** ^1^ School of Dentistry The University of Queensland Herston Queensland Australia; ^2^ Department of Periodontics and Implantology Griffith University, School of Medicine and Dentistry Queensland Australia

**Keywords:** COVID‐19, dentistry, depression, anxiety, stress

## Abstract

**Aims:**

The dentistry program is extremely demanding mentally and physically. Consequently, it can induce high levels of stress, anxiety and depression in students. There is some research in measuring these ramifications on dental students, but they lack the influence of the COVID‐19 pandemic which has brought about many fundamental changes to the curriculum of dental students. The aim of this study was to assess dental students' mental health through the Depression, Anxiety and Stress Scale (DASS‐21) during the COVID‐19 pandemic in an Australian dental School.

**Methods:**

The Bachelor of Dental Science (Honours) students enrolled in years 2–5 were requested to complete an online questionnaire that included the DASS‐21 and additional questions regarding the impact of COVID‐19 on quality of life. Jamovi was utilized to conduct descriptive data analysis.

**Results:**

179 students completed the survey with 81 males (45%) and 98 females (55%). 70.4% of the participants described elevated anxiety for the health of their loved ones and themselves during the COVID‐19 pandemic. The overall mean DASS‐21 scores were 7.1 (5.07) in depression, 4.9 (4.00) in anxiety and 6.5 (4.32) in stress.

**Conclusions:**

The results indicate that the COVID‐19 pandemic negatively impacted the mental health of dental students. While further research is still required, it is important for universities to recognize how this pandemic affected the mental wellbeing of students so that they can implement appropriate support programs and improve dental education.

**Clinical relevance:**

The COVID‐19 pandemic has had a profound impact on social and mental health worldwide, and dental students are notably affected. Recognized as a psychological risk factor, COVID‐19 has been linked to an increased rate of suicidal deaths. This paper underlines the importance of recognizing the full scope of the pandemic's impact on dental students, including their views and the potential effects on their physical and mental health. The study indicates a pressing need for more robust support from the organizations to improve the mental health and overall well‐being of dental students.

**Abbreviation and acronym**
UQ: University of Queensland

## 1 INTRODUCTION

Dentistry has long been considered a demanding profession. Likewise, dental school is a challenging university program. Throughout the dental school program, students are required to master a vast degree of clinical skills requiring complex manual dexterity, balancing this with a strenuous academic curriculum.^1^ These skills are highly difficult to learn and take a considerable time commitment to grasp. Students from The University of Queensland (UQ) can expect up to 30 h a week in workload. These challenges are compounded for students studying abroad or interstate, who might have to adjust to being away from family and adapt to living in a new environment. Sources for stress vary as students' progress through the degree.^1^ For instance, during pre‐clinical years, stress might originate from the heavy academic workload.^1^ In contrast, during clinical years, sources of stress might include having to learn clinical procedures, deal with challenging patients and be able to provide complex treatment in a restricted time limit.^2^ Dental students in university have an elevated risk of suffering from mental health issues.^3^ Prior literature has recognized the university environment and increasing workload throughout the degree as a major source of stress for this population.^3^ Dentistry is an especially physically and mentally challenging program. Inability to acquire the required competence in these skills results in failure to progress in the course which can be detrimental on the mental health of dental students. Overall, the dental profession is associated with a greater risk of stress, depression and suicide, and dental students experience this as well.^4^

On 31st December 2019 a novel coronavirus (COVID‐19) was detected in Wuhan, China. The virus was first reported outside of China on 13th January 2020 in Thailand and since then has rapidly spread across the globe. Consequently, the World Health Organization (WHO) announced the COVID‐19 outbreak as a global pandemic on 11th March 2020.^5^ The COVID‐19 pandemic has impacted the livelihood, health and communities of billions of people globally (WHO). This widespread infection presented an array of unprecedented challenges that the world was not prepared to face. Stress arose from a newfound risk to one's health and its consequences on a multitude of aspects in one's life. The understanding of the virus was changing constantly at a rapid rate and thus the responses were frequently evolving. Responses such as restricting travel, mandatory lockdowns and widespread social distancing were implemented. The first reports of the virus within Australia came on 25th January 2020. Consequently, UQ cancelled in‐person lectures, preclinical activities and clinical practice from 26th March 2020 and onwards. The state of Queensland in Australia entered lockdown for the first time on 30th March 2020.^6^ Queensland started its easing of restrictions on 8th May 2020. By 3rd of July 2020, all restrictions were lifted in Queensland. The first day of semester 2 with all in‐person classes was resumed on 3rd August 2020. Spontaneous lockdowns were also implemented throughout 2021 on multiple occasions as COVID‐19 cases started to fluctuate, prolonging the ramifications of COVID‐19. As a result of this chaos and uncertainty, the coronavirus provided many obstacles for dental students to stay focused and engaged in their academic endeavours. Additionally, with health authorities implementing strict restrictions involving social isolation and social distancing, many students were isolated from family and friends, minimizing the amount of overall social support available to help them through their struggles during the COVID‐19 pandemic.^7^

Experiencing stress is natural for humans and in some cases is beneficial as it might act as a motivating factor to achieve and perform at a higher level.^8^ However, it has been observed that untreated high levels of chronic stress can be detrimental as it might result in anxiety, depression and physical manifestations that affect overall health.^8^ As dental students have been shown to exhibit high levels of stress, the consequences of this might span from reduced academic performance or difficulty interacting with patients due to lack of motivation and concentration, to more severe repercussions such as self‐harm and suicide.^9^ A previous study carried out by Stormon *et al*. used the DASS‐21 to measure the mental wellbeing of dental students in an Australia school prior to COVID‐19.^10^ Due to the potential for increased levels of stress stemming from the COVID‐19 pandemic, it was important to identify the causes of stress among this population to help reduce the prevalence of mental disorders. Thus, the aim of this study was to assess the impact of COVID‐19 and the stress‐related factors involved during the pandemic on the mental wellbeing of dentistry students.

## 2 METHODOLOGY

### 2.1 Study design and participants

This cross‐sectional questionnaire study involved the second to fifth years of UQ Bachelor of Dental Science (Honours). The study was approved by the Institutional Human Research Ethics committee (Ethics Approval Number: 2021/HE001720). All students from the second to fifth year cohorts were invited to participate in this study. Participants provided consent to participate prior to the commencement of the survey. Those who refused to participate were excluded from the study.

### 2.2 Outcome measures and survey tool

This study involved the completion of one survey during semester 2 of the 2021 academic year whereby the students reflected on their mental health and other associated factors from March 2020 to July 2020. The survey consisted of three parts: Part A included demographic questions; Part B had questions specific about the impact of the COVID‐19 pandemic on their lives; and Part C contained the Depression Anxiety Stress Scale (DASS‐21). General questions were asked to determine the demographic information of the participants. These questions assessed their age, gender, year level, international or domestic student status, marital status, living arrangements, whether they are enrolled at university internally or externally, language spoken at home, access to a general practitioner (GP), and whether or not studying dentistry was their number one career preference. These questions are related to factors that might be potential sources of stress and will therefore assist in identifying trends in the data collected.

The DASS‐21 evaluated anxiety, depression and stress through a verified questionnaire and in a quantitative manner.^11^ This scale was constructed by Lovibond in 1995 and has been used by psychologists and clinicians to help screen for symptoms at various levels of depression, anxiety, and stress.^12^ The DASS‐21 has 21 questions that were split into three groups of seven questions each. The three groups were aimed to measure depression, anxiety and stress. Each question was rated on a 4‐point Likert scale that goes from 0 (does not apply at all) to 3 (applies very much or most of the time). The higher the score, the higher the level of depression, anxiety and stress. The total scores for depression, anxiety and stress were determined by calculating the sum of the scores for each relevant item. The DASS‐21 scores were categorized on a range of severity from normal to extremely severe (Table 1).

**Table 1:** The cut‐off scores for the severity ranks of the DASS‐21^11^

Severity rank	Depression	Anxiety	Stress
Normal	0–4	0–3	0–7
Mild	5–6	4–5	8–9
Moderate	7–10	6–7	10–12
Severe	11–13	8–9	13–16
Extremely severe	14+	10+	17+

### 2.3 Statistical analysis

The data were tabulated on Microsoft Excel spreadsheet (Version 2008) and then imported into Jamovi (Version 1.6.3) Statistics for Windows (Microsoft, Redmond, Washington, USA) for descriptive analysis and GraphPad PRISM 9.0 software (GraphPad Software, San Diego, Calif., USA) for collation and creation of appropriate graphs. The responses were summarized, and comparisons were made. The output of data was presented in a table format (total responses and percentage) as well as a graphical format. Specific data analysis tests performed included descriptive statistics, such as mean and standard deviation. The comparison of depression, stress and anxiety with different year level, gender, location classes, and those with dentistry as their first preference was done student *t*‐test in GraphPad PRISM.

## 3 RESULTS

### 3.1 Demographics

The total number of dental students between the years 2–5 was 313 and of that, 228 voluntarily participated in the study. There were more female participants (54.7%) compared to male participants (45.3%), 179 of these students completed the survey in its entirety, thus achieving a 57% rate of participation to completion. The demographic data shows that the majority of participants were aged between 20 and 29, domestic students (51.4%), unmarried (92.7%), spoke English fluently (78.7%), lived with roommates (46.4%), had access to a GP (65.4%), selected to study dentistry as their first preference (84.4%) and attended class internally (on‐shore) during the year 2020 (93.9%). The majority of students that were off‐shore (international students) belonged to the 2nd year level (Table 2).

**Table 2:** Dental student demographics

Demographics	*n* (%)
Age	
≤19	10 (5.6)
20–29	162 (90.5)
30≥	7 (3.9)
Gender	
Male	81 (45.3)
Female	98 (54.7)
BDSc (Hons) year level	
Year 2	42 (23.5)
Year 3	44 (24.6)
Year 4	55 (30.7)
Year 5	38 (21.2)
Student status	
Domestic	92 (51.4)
International	87 (48.6)
Marital status	
Single	166 (92.7)
Married	13 (7.2)
Living arrangements	
At home with family	41 (22.9)
With roommates	83 (46.4)
Live alone	43 (24.0)
Living at college	5 (2.8)
Other	7 (3.9)
Location of attendance for BDSc (Hons) classes	
Internally (Brisbane, Australia)	168 (93.9)
Externally (off‐shore)	11 (6.1)
What language(s) are you fluent in	
English	141 (78.7)
Other	38 (21.2)
Access to usual GP	
Yes	117 (65.4)
No	62 (34.6)
Dentistry first preference	
Yes	151 (84.4)
No	28 (15.6)

### 3.2 Dental students personal feelings on impact of COVID‐19

**Fig. 1:**
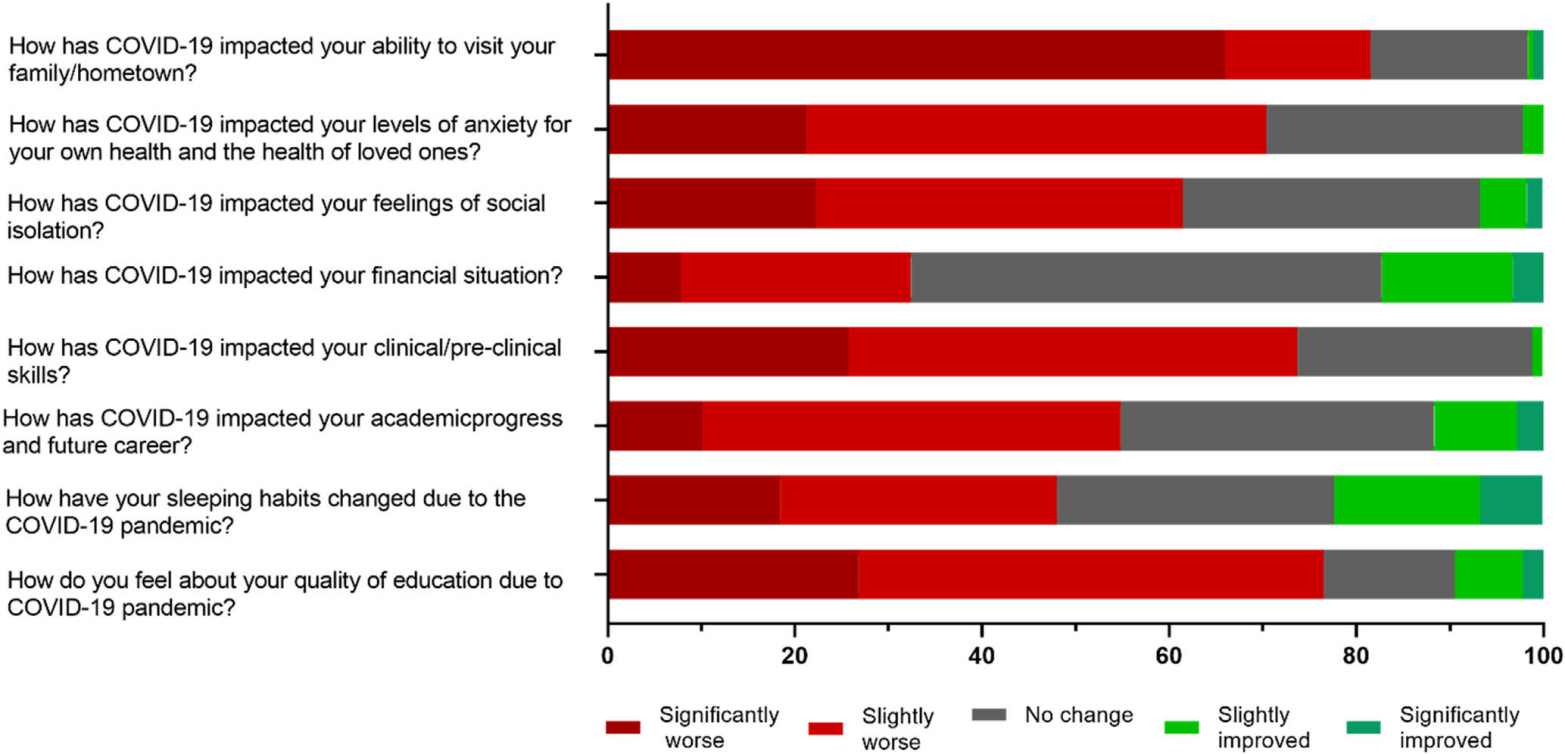
Students personal feelings on impact of COVID‐19 on daily routine, family and education.

The summary of the personal feelings of dental students on the impact of COVID‐19 pandemic on their education, lifestyle routine and family/social life is presented in Fig. 1. It was found that 76.5% of the total population felt that the quality of education was worse due to COVID‐19 (Table 2). The majority of the dental students (73.4%) felt that their clinical and pre‐clinical skill were worse due to the impact of COVID‐19. Additionally, 44.7% reported a slightly worse impact from the COVID‐19 pandemic on their academic progress and future career. Regarding daily routine, 59.2% of students displayed either no change (29.6%) or slightly worse (29.6%) change in their sleep routine due to the COVID‐19 pandemic. Furthermore, 65.9% reported that the COVID‐19 pandemic had a significantly worse impact in their ability for them to visit their family/hometown. Concerning the impact of the COVID‐19 pandemic on the financial situation of the dental students, it was found that 49.7% had some form of financial hardship in this area. It was also found that 70.4% of the participants felt that their levels of anxiety increased.

### 3.3 Depression, anxiety and stress mean scores

**Fig. 2:**
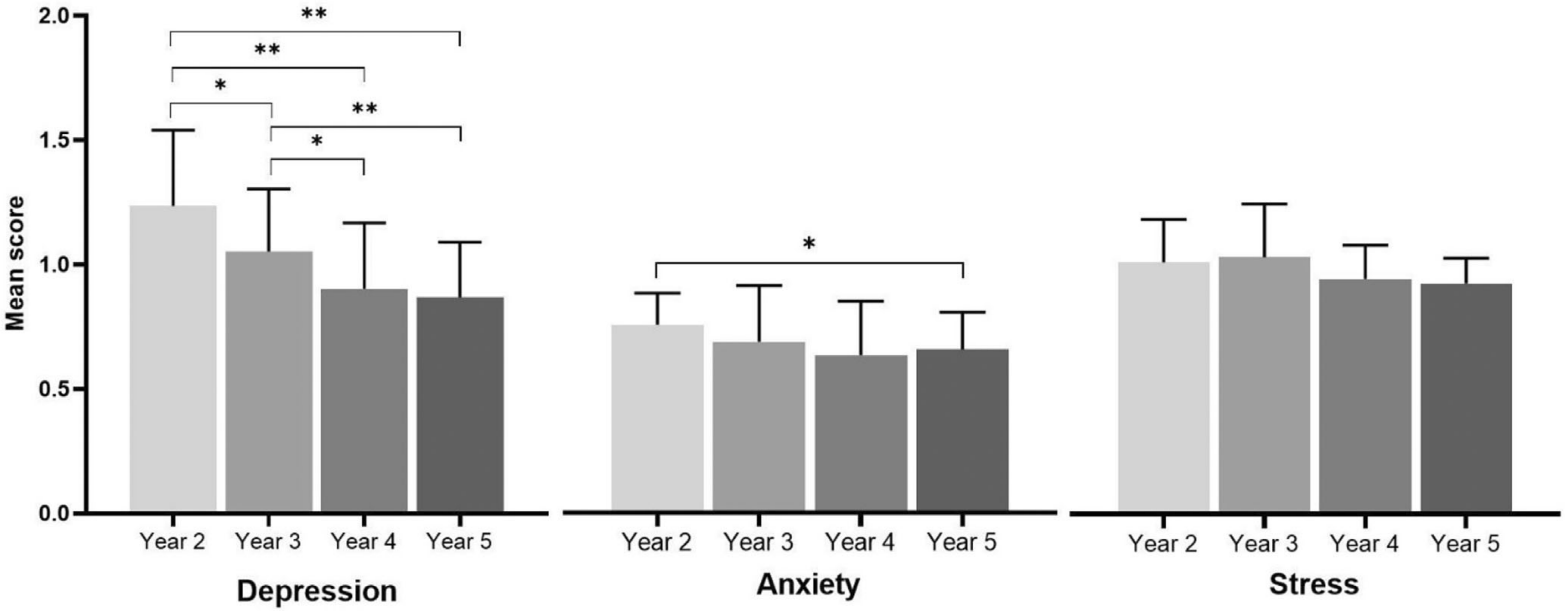
Mean DASS‐21 scores per category. Results expressed as mean with 95% confidence interval. *P ≤ 0.05; **P ≤ 0.005.

Overall, the mean depression, anxiety and stress scores were 7.1 (SD: 5.07), 4.9 (SD: 4.00) and 6.5 (SD: 4.32) respectively. According to the DASS‐21 severity scale these scores depict overall moderate depression, mild anxiety and normal stress within the total population. When evaluating these scores across the different years, it was found that the second‐year level cohort had the highest mean score for depression and anxiety, whereas the 3rd year cohort had the highest mean score for stress (Fig. 2). The individual DASS‐21 item seen with the highest mean score across all year levels was *“I found it difficult to work up the initiative to do things”* (Table 3). Overall, the depression, anxiety and stress scores decrease as the year level increases for the student, with the only outlier being the stress of the third years being slightly higher than the second years.

**Table 3:** Mean DASS‐21 scores per question

DASS‐21	Overall, Mean (SD)	Year 2, Mean (SD)	Year 3, Mean (SD)	Year 4, Mean (SD)	Year 5, Mean (SD)
DEPRESSION					
I couldn't seem to experience any positive feeling at all	0.93 (0.80)	1.10 (0.88)	0.96 (0.71)	0.93 (0.77)	0.74 (0.83)
I found it difficult to work up the initiative to do things	1.56 (0.87)	1.86 (0.90)	1.61 (0.78)	1.44 (0.88)	1.34 (0.85)
I felt that I had nothing to look forward to	1.02 (0.95)	1.31 (0.92)	1.05 (1.10)	0.87 (0.80)	0.87 (0.94)
I felt down‐hearted and blue	1.08 (0.82)	1.36 (0.79)	1.02 (0.88)	1.02 (0.76)	0.92 (0.85)
I was unable to become enthusiastic about anything	1.02 (0.92)	1.21 (0.84)	1.11 (1.10)	0.86 (0.73)	0.92 (0.97)
I felt I wasn't worth much as a person	0.73 (0.85)	0.93 (0.78)	0.80 (0.98)	0.58 (0.71)	0.63 (0.91)
I felt that life was meaningless	0.74 (0.87)	0.88 (0.80)	0.82 (1.02)	0.62 (0.76)	0.66 (0.91)
Anxiety					
I was aware of dryness of my mouth	0.71 (0.84)	0.71 (0.81)	0.68 (0.88)	0.71 (0.81)	0.74 (0.92)
I experienced breathing difficulty (eg, excessively rapid breathing, breathlessness in absence of physical exertion)	0.45 (0.65)	0.55 (0.63)	0.41 (0.69)	0.44 (0.63)	0.40 (0.68)
I experienced trembling (eg, in the hands)	0.51 (0.74)	0.60 (0.73)	0.50 (0.73)	0.49 (0.72)	0.47 (0.80)
I was worried about situations in which I might panic and make a fool of myself	0.98 (0.91)	0.88 (0.92)	1.11 (0.92)	1.04 (0.92)	0.84 (0.86)
I felt I was close to panic	0.69 (0.79)	0.88 (0.86)	0.68 (0.80)	0.56 (0.71)	0.68 (0.81)
I was aware of action of my heart in absence of physical exertion (eg, sense of heart rate increase, heart missing a beat)	0.58 (0.74)	0.71 (0.89)	0.50 (0.73)	0.53 (0.60)	0.61 (0.76)
I felt scared without any good reason	0.55 (0.73)	0.64 (0.73)	0.64 (0.87)	0.40 (0.56)	0.58 (0.76)
Stress					
I found it hard to wind down	1.09 (0.82)	1.17 (0.82)	1.18 (0.82)	1.09 (0.80)	0.90 (0.86)
I tended to over‐react to situations	1.01 (0.80)	1.10 (0.85)	1.16 (0.81)	0.96 (0.69)	0.79 (0.84)
I felt that I was using a lot of nervous energy	0.93 (0.81)	0.98 (0.87)	1.00 (0.86)	0.82 (0.64)	0.95 (0.90)
I found myself getting agitated	0.93 (0.81)	0.86 (0.78)	1.05 (0.89)	0.96 (0.79)	0.82 (0.77)
I found it difficult to relax	1.04 (0.87)	1.10 (0.96)	1.07 (0.93)	0.96 (0.79)	1.05 (0.84)
I was intolerant of anything that kept me from getting on with what I was doing	0.84 (0.81)	0.81 (0.78)	0.84 (0.86)	0.82 (0.77)	0.90 (0.86)
I felt that I was rather touchy	0.65 (0.68)	0.69 (0.60)	0.55 (0.73)	0.66 (0.62)	0.74 (0.80)

When comparing the DASS‐21 scores between males and females, females noted higher mean scores in depression, anxiety and stress at 7.45 (SD 4.72), 5.35 (SD 4.12) and 7.43 (SD 4.32) respectively. In contrast, males scored 6.60 (SD 5.45), 4.44 (SD 3.80) and 5.34 (SD 4.04) in those categories. Dental students who did not select dentistry as their first preference for university programs showed a higher mean overall depression of 7.82 (SD 3.93), anxiety 5.61 (SD 3.60) and stress 6.57 (SD 3.65). It was also observed that international students had mean depression 6.76 (SD 4.77), anxiety 4.84 (SD 3.55) and stress 6.41 (SD 4.1) scores which were less than that of domestic students 7.36 (SD 5.34), 5.03 (SD 4.40) and 6.57 (SD 4.53), correspondingly.

## 4 DISCUSSION

The study revealed that the participating dental students experienced moderate depression, mild anxiety and normal stress levels according to the DASS‐21 severity ranking. Furthermore, the levels of depression, anxiety and stress assessed in this study were compared to that of a previous study carried out at UQ SoD, who surveyed the same year levels of students at the same dental school using the DASS‐21 survey pre‐COVID‐19.^10^ Additionally, each of the three categories – depression, anxiety and stress – included in the DASS‐21 survey done during the pandemic had higher overall mean scores compared to their baseline group, further revealing a diminishing level of mental wellbeing in this student sample compared to previous student samples.^10^ One reason for this finding could be due to impacts of COVID‐19, as it was found in another paper that depression in dental students were recorded to be 1.78 times more prevalent if their relatives had had been infected with COVID‐19.^13^ Fear for the health of family members compounded with extended periods of time spent in isolation could be large contributing factors to the rise in rates of depression.^13^ In addition, it has been seen that young adults who have undergone quarantine and social isolation restrictions might be more prone to experiencing higher levels of anxiety and depression.^14^ This might be due to a greater need to socialize and establish a network of relationships outside of their homes compared to adults.^14^ One similar finding observed in both the Stormon *et al*. paper and this study was that the highest individual scoring item in both participating groups was *“I found it difficult to work up the initiative to do things”*. This finding was more severe in this study and this might be due to the dramatic and swift change from an in‐person to online learning delivery during COVID‐19, forcing students to adapt and change to a new dynamic approach to learning and teaching.^10^ This was also seen in another cross‐sectional study in which 97.7% of dental students in a Malaysian cohort found it difficult to focus on their academic tasks and had a lack of initiative during the pandemic era.^15^

The pre‐COVID‐19 baseline scores from the Stormon *et al*. study can be compared to data collected in this study to assess the impact of COVID‐19 on mental wellbeing.^10^ Their study reported a mean depression score of 4.7 (SD 3.9), compared to a score of 7.1 (SD 5.1) in this study. The mean anxiety score in their study was observed to be 4.3 (SD 3.2), in comparison to a score of 4.9 (SD 4.0) during COVID‐19. Furthermore, the mean stress score observed in their 2017 cohort was 5.5 (SD 3.7), whereas the mean stress score for this study was 6.5 (SD 4.3). The comparison to the baseline 2017 cohort in their study showed that in all three categories of depression, anxiety and stress, there was an increase in the mean scores in the participants of this study. In addition, as mentioned above, the DASS‐21 survey question with the highest mean score 1.56 (SD 0.87) in this study was *“I found it difficult to work up the initiative to do things”*. This coincided with the 2017 cohort as it was this same question that had the highest mean score 1.1 (SD 0.8).^10^

The study also showed that pre‐clinical years (year 2–3) had significantly higher levels of depression (*P =* 0.0001, year 2 and 5) anxiety (*P =* 0.01, year 2 and 5) and stress compared to clinical years (year 4–5). Levels of depression (8.64) and anxiety (5.64) were highest among second year students, whereas third year students reported the highest levels of stress (6.84). The second‐year students, who were in their first year at the start of the pandemic, had never experienced university before, thus the adjustment to the workload might contribute to their poorer mental health. The third‐year students were in second year at the start of the pandemic, which is the year during which they develop their hands on skills in pre‐clinic. Thus, the cancellation of pre‐clinical time while still requiring passing grades could manifest as elevated stress. Therefore, the challenges brought by the pandemic compounded with the fear of exams and failure, as well as inexperience in adversity, might have resulted in pre‐clinical students suffering from elevated depression, anxiety and stress.^16^ It was also found that fifth year students had the lowest scores in depression (6.08), anxiety (4.61) and stress (6.13). The reason for this trend could be due to higher levels of maturity and experience in the university environment and thus greater ability to adjust to adversity for the clinical year students compared to pre‐clinical year students who might lack resilience to the challenges of dentistry and university.

Additionally, 76.5% of the participants felt that the quality of education they were receiving was worse due to COVID‐19 and the changes it brought to the curriculum. Face‐to‐face lectures and learning activities were replaced with online learning and the implementation of lockdowns and strict restrictions due to the pandemic resulted in significant reduction in clinical and preclinical exposure. Consequently, the majority of participants (73.4%) felt that their clinical and pre‐clinical skills were negatively impacted. A study carried out at the University of Otago assessed dental students and dental staff perceptions of the educational impact during COVID‐19 found that the majority of students and staff felt that the closure of teaching clinics would cause ‘extreme impact’ on students' clinical competence.^17^ As dentistry is a profession that requires proficient hand skills and mastering clinical skills to provide appropriate care for patients, it is more burdensome on dental students to reach that level of skill and acquire that ability clinically when there is a reduced amount of access and clinical time to do so due to COVID‐19 restrictions. Consequently, students are under more pressure to attain those skillsets in a shorter timeframe and therefore are more likely to suffer from higher levels of stress, anxiety and depression.

The majority of participants (70.4%) felt that their levels of anxiety for their own health and the health of loved ones were worse during the COVID‐19 pandemic. This can be attributed to the abrupt changes caused by the pandemic as many studies have associated elevated anxiety levels with traumatic events that are unpredictable or long lasting such as the COVID‐19 pandemic.^18^ Participants might have had increased concerns for relatives in vulnerable populations such as the pregnant, elderly or immunocompromised, who are more likely to suffer from complications due to the virus.^18^ Furthermore, it was found that 43% of college students displayed worry for the wellbeing of family members in those vulnerable categories.^18^ A study from Alrashed *et al*. investigated this correlation and found that feelings of hopelessness were 2.4 times higher in dental students who themselves contracted or had close relatives infected with the virus.^13^

One of the many restrictions introduced as a result of the pandemic was travel restrictions. Both international and domestic travel were banned unless there were exceptional circumstances. The travel ban impacted a large proportion of dental students. According to the survey, 81.5% of participants responded saying that their ability to visit their family or hometown was negatively impacted. As a result, these students might have lacked an immediate social support network from family and friends during this period of time, contributing to a greater prevalence of social isolation, and consequently, elevated rates of depression, anxiety and stress.^19^ This research indicated that international students had higher severity DASS‐21 scores compared to domestic students. Other similar studies have also found that university students studying away from home had higher DASS‐21 severity scores, highlighting the possible impact of being away from family on the mental wellbeing of university students studying abroad.^18^, ^19^

This study also found that the DASS‐21 scores were higher in female dental students compared to male dental students. Similar findings were found in the Stormon *et al*. paper which showed that females had a greater risk compared to males for more severe DASS‐21 scores.^10^ Moreover, in a paper done by Lestari *et al*., it was found that the female dental students experienced a greater level of stress in comparison to their male peers.^15^ This could be due to gender‐related stressors, coping mechanisms during COVID‐19 pandemic, workplace dynamics, and patient interactions. Despite survey responses being anonymous, females have an increased inclination to be more open about their emotions compared to their males counterparts, which could have been elevated when completing the survey.^15^ Participants were asked whether or not studying dentistry was their first preference for a university degree. Most students (84.4%) selected dentistry as their first preference, whereas a minority of students (15.6%) did not. However, it was observed that those students who did not choose dentistry as their ideal career preference had higher levels of depression 7.82 (SD 3.93), anxiety 5.61 (SD 3.60) and stress 6.57 (SD 3.65), however it was not statistically significant. A similar trend was observed in the study by Stormon *et al*. where the 12.4% of students who did not select dentistry as their first choice had higher DASS‐21 scores.^10^ These students might have chosen to study dentistry for reasons such as status, high income, family or peer pressure, or might not have achieved the academic requirements to enter into their preferred university degree. Studies have shown that such students who do not possess the desire to become dentists might suffer from a lack of passion and determination in their academic endeavours. Consequently, these students are more prone to higher levels of stress, anxiety and depression.^10^

A cross‐sectional study from Italy that assessed the stress and anxiety among dental students facing COVID‐19 also found that the students experienced negatively in their study career and mental health.^20^ In another study, over 11% of dental and dental hygiene students reported that their career plans have changed due to COVID‐19.^21^ Those students showed significantly higher perceived levels of stress due to COIVD outbreak. In a recent cohort study (4047 students) investigating the mental health of local and international students attending university, Russell and co‐workers found that the COVID outbreak negatively impacted international students compared to local students and reported inequalities on several bases.^22^

This study included several limitations. The first of which is that the questionnaire was carried out as a retrospective survey that collected responses from participants requiring them to reflect back on their mental wellbeing and experiences during the peak of the pandemic. This limited the students from having a present or more recent memory of how they felt during that specific time period, which might have caused recall bias in the data. In addition, this study assessed dental students from only one Australian dental school. In addition, every individual might respond differently when faced with stress and adversity in day‐to‐day life. One should consider that each student differs in their level of psychological and emotional maturity, genetics, personality and life experiences, influencing their ability to cope with hardships. Finally, the study did not ask questions regarding the participants previous history of depression or anxiety. Although this study has its limitations, the findings still provide insight in this area of research on mental wellbeing and COVID‐19 in dental students, however further research is needed to better establish and support the finding of this paper.

Whilst the COVID‐19 pandemic presented an array of difficulties for students, the same can be said about the university. University staff played a vital role in implementing strategies to minimize stress‐levels for the student population during COVID‐19. Introduction of flexible delivery of courses with new online lectures and teaching modules were provided to students. New teaching strategies have been successfully explored to improve students' learning experiences. Similarly, new research should focus on novel pedagogical designs and how students can be prepared to face such challenging circumstances. Furthermore, continuous timetable adjustments were made to cater to students' needs and there was increased responsibility on staff to adjust course requirements and assessments in a short time period. As a result, it is with equal importance for future studies to investigate the mental health and wellbeing of university staff during the COVID‐19 era.

## 5 CONCLUSIONS

This study investigated the mental wellbeing of dental students during the COVID‐19 pandemic. University students, particularly dental students, already experience high levels of stress, anxiety and depression due to the taxing nature of the degree. The COVID‐19 pandemic introduced a multitude of additional challenges which were considered in this study. Using the DASS‐21 tool, levels of stress, anxiety and depression were measured, and it was found that in all three categories, scores were higher than those found in similar studies discussed that were done prior to COVID‐19. The results indicate that the COVID‐19 pandemic negatively impacted the mental health of dental students. Although further research is still required, this paper has provided insight on the importance of recognizing the factors that contribute to poor mental health for dental students as well as future opportunities for dental education development and support programs, thus allowing for future positive mental health outcomes.

## AUTHOR CONTRIBUTIONS

**S Akhtar:** Investigation; writing – original draft; methodology; formal analysis. **J Tissainayagam:** Investigation; writing – original draft; methodology; formal analysis. **J Lo:** Investigation; writing – original draft; formal analysis. **A Siddiqi:** Methodology; writing – review and editing; project administration; resources. **S Zafar:** Conceptualization; investigation; writing – review and editing; methodology; formal analysis; supervision.
